# Quantitative Systems Pharmacology Analysis of Hippocampal Volume Trajectory by APOE Genotype and Neuroprotective Effects of Valiltramiprosate/ALZ‐801 in Early AD

**DOI:** 10.1002/alz70861_108550

**Published:** 2025-12-23

**Authors:** Hugo Geerts, Shaina M Short, Jeremy Yu, Jean Schaefer, Susan Abushakra, Aidan Power, Martin Tolar, John A. Hey

**Affiliations:** ^1^ In Silico Biosciences, Berwyn, PA USA; ^2^ Certara Predictive Technologies, Berwyn, PA USA; ^3^ Certara Predictive Technologies, Park City, UT USA; ^4^ Alzheon, Framingham, MA USA; ^5^ Alzheon, Inc., Framingham, MA USA

## Abstract

**Background:**

Valiltramiprosate is an oral, small‐molecule inhibitor of β‐amyloid oligomer formation in late‐stage development as a disease‐modifying therapy for AD. We provide a quantitative systems pharmacology analysis of the natural AD trajectory of hippocampal volume changes by APOE genotype and the neuroprotective effect of valiltramiprosate in Early AD.

**Method:**

A novel QSP model for natural AD trajectory of hippocampal volume was developed, integrating factors of age, non‐amyloid pathology, amyloid oligomers, protofibrils and plaques, ARIA‐E, clearance and microglia activation. The model applied longitudinal observational and interventional data from the lecanemab CLARITY and donanemab TRAILBLAZER clinical trials. Impact of APOE genotype was applied based on its differential Aβ synthesis and clearance. The analysis of valiltramiprosate effect was based on mechanism of action on inhibition of Aβ oligomer formation and an effect as an APOE4 structural corrector.

**Result:**

The QSP trajectory model of hippocampal volume in AD exhibited pronounced genotype differences in disease progression, with APOE4/4 >> APOE3/4 > APOE3/3. APOE4/4 AD subjects exhibit a greater degree of disease acceleration in Aβ pathology and hippocampal atrophy compared to APOE3/4 and APOE3/3, including an earlier onset (∼4‐year leftward shift) and more rapid decline consistent with an earlier manifestation of pathologies prior to clinical decline. QSP analysis of valiltramiprosate pharmacology demonstrates a substantial reduction of neurotoxic oligomers and protofibrils, leading to a reduction of hippocampal atrophy that is exposure, age and disease‐stage dependent. Due to its anti‐aggregation MOA upstream in the amyloid cascade, there is minimal effect on plaque deposition and thereby no ARIA liability, consistent with the safety results from APOLLOE4 Phase 3 study evaluating valiltramiprosate.

**Conclusion:**

QSP analysis supports the hypothesis that acceleration of Aβ aggregation is the initial step in Early AD triggering an increase in toxic oligomers that drives AD neurodegeneration, measured by hippocampal atrophy, followed by subsequent rapid cognitive decline. Valiltramiprosate intervention in Early AD produces an exposure, age and stage of disease‐dependent reduction in hippocampal atrophy in all APOE genotypes, with the magnitude of benefit greatest at early symptomatic disease stage. The neuroprotective effect is driven by an exposure‐related reduction of Aβ oligomers and protofibrils that blunts the pathological amyloid cascade in AD.

